# Validation of a 3D planning tool for glenoid bone loss evaluation in revision anatomic total shoulder arthroplasty

**DOI:** 10.1186/s12891-026-10087-6

**Published:** 2026-06-18

**Authors:** Mara Warnhoff, Florian Freislederer, Julia Högl, Daniela Brune, Asimina Lazaridou, Markus Scheibel

**Affiliations:** 1 Department of Shoulder and Elbow Surgery, Schulthess Clinic, Lengghalde 2, Zurich, 8008 Switzerland; 2 Center for Musculoskeletal Surgery, Charité-Universitaetsmedizin Berlin, Berlin, Germany

**Keywords:** Shoulder arthroplasty, Revision, Preoperative planning software, Glenoid defect

## Abstract

**Background:**

Accurate preoperative assessment of glenoid bone loss is critical in revision shoulder arthroplasty. The aim of this study was to evaluate the reliability of the Blueprint Revision 3D-CT planning software (Stryker, Kalamazoo, MI, USA) by comparing preoperative reconstructions with intraoperative findings. Agreement between preoperative and intraoperative assessments was analyzed for glenoid containment status, comparing 2D-CT and 3D reconstructed images derived from CT imaging (3D-CT) with intraoperative findings.

**Methods:**

Forty-seven patients undergoing revision of the same stemless anatomic total shoulder arthroplasty with a cemented polyethylene glenoid component were included in this analysis. Glenoid bone defects were classified using the established systems according to Antuña and Williams-Iannotti. Defect containment (contained vs. uncontained) was assessed preoperatively using 3D-CT and 2D-CT imaging and compared with intraoperative findings. Agreement between different imaging methods was evaluated using Cohen’s kappa coefficient (κ).

**Results:**

The most common indication for revision was glenoid loosening (79%), with a mean time to revision of 7 ± 3 years. Intraoperative evaluation classified 68.1% of glenoid defects as contained, which was correctly depicted in 80.9% of 3D-CT and 65.9% of 2D-CT. Agreement between 3D-CT and intraoperative findings was moderate (κ = 0.58, 80.9%) and outperformed 2D-CT (κ = 0.37, 65.9%). Agreement between 3D-CT and 2D-CT was also moderate (κ = 0.48, 72.3%).

**Conclusion:**

3D-CT–based preoperative planning using Blueprint Revision demonstrates higher agreement with intraoperative findings compared to traditional 2D imaging. These findings support the value of advanced 3D planning tools in evaluating glenoid containment and guiding surgical decision-making in revision shoulder arthroplasty.

## Introduction

With the increasing number of shoulder arthroplasty procedures [1–6], the number of revision surgeries has likewise risen [7–10], adding considerable clinical complexity for orthopaedic surgeons. One of the most critical issues in revision anatomic total shoulder arthroplasty (aTSA) is the presence of glenoid bone defects, which complicate both surgical strategy and execution [11–13]. Metal artifacts and residual implants often obscure these defects, reducing imaging quality and hindering accurate preoperative assessment.

While digital planning tools have become standard in primary shoulder arthroplasty [14, 15], comparable solutions for revision procedures have only recently emerged. Until recently, surgical planning for glenoid reconstruction in revision cases often remained uncertain until the intraoperative stage. The development of advanced 3D software, such as Blueprint Revision (Stryker, Kalamazoo, MI, USA), offers a potential solution by enabling 3D reconstruction and visualization of glenoid defects despite the challenges posed by artifacts and previous implants, yet data regarding its reliability remain limited [16].

This study evaluated the accuracy of 3D reconstructed images derived from CT imaging using the Blueprint Revision planning software by comparing preoperative reconstructions with intraoperative findings in revision of a single aTSA implant (Eclipse Stemless Shoulder Arthroplasty System with a cemented pegged all-polyethylene glenoid component; Arthrex, Naples, FL, USA). Specifically, it aimed to assess concordance in defect containment (contained vs. uncontained) between intraoperative evaluation and preoperative imaging using both 2D and 3D CT modalities.

## Materials & methods

Study procedures received approval by the local Institutional Review Board (approval number: KEK-ZH-NR. 2014 − 0483). All procedures performed were in accordance with the ethical standards of the institutional and/or national research committee and with the 1964 Helsinki declaration and its later amendments. Written informed consent was obtained from all participants.

This level III retrospective cohort study included patients identified from our institutional database who underwent revision surgery after aTSA with cemented pegged polyethylene glenoid components (Arthrex, Naples, FL, USA). As this was a retrospective exploratory cohort study, no a priori sample size calculation was performed. Patients were included in the study if 3D preoperative planning using Blueprint software version 4.0.1 (Stryker, Kalamazoo, MI, USA) had been performed prior to revision. For patients without prior planning, retrospective eligibility for software-based reconstruction was assessed. Inclusion criteria required preoperative imaging with available CT datasets. In cases undergoing bone scintigraphy, hybrid SPECT/CT imaging was available, and the CT component was used for analysis. Therefore, all included patients had preoperative CT-based imaging suitable for both 2D and 3D assessment, as well as intraoperative photographic imaging of the glenoid defect or description of the containment of the defect in the surgical protocol. If both criteria were met, the CT datasets in DICOM format were uploaded into the Blueprint Revision software. Company engineers manually performed slice-by-slice segmentation of the proximal humerus and glenoid region, while metallic implants and imaging artifacts were identified and excluded. Following segmentation, the datasets were processed to generate a three-dimensional reconstruction for preoperative assessment within the software environment. Glenoid defects were preoperatively categorized on 2D and 3D-CT scans using existing classifications according to Antuña and Williams-Iannotti [17, 18]. The Antuña classification [17], describes intraoperative bone loss by location (central, peripheral, or combined) and severity (mild, moderate, severe), later expanded to differentiate centric and eccentric defects based on vault destruction and extent. The Williams-Iannotti [18] classification evaluates glenoid loss by the integrity of the subchondral bone, vault, and rim. Although each system offers specific advantages, neither fully captures all factors relevant to implant fixation in complex revision cases [17, 18].

For included patients, intraoperative containment status was determined based on standardized review of operative photographs and operative reports. A contained defect was defined as preservation of sufficient circumferential bony support, whereas uncontained defects demonstrated segmental loss of the glenoid rim. When both photographic and written documentation were available, photographic documentation was prioritized.

**Fig. 1:**
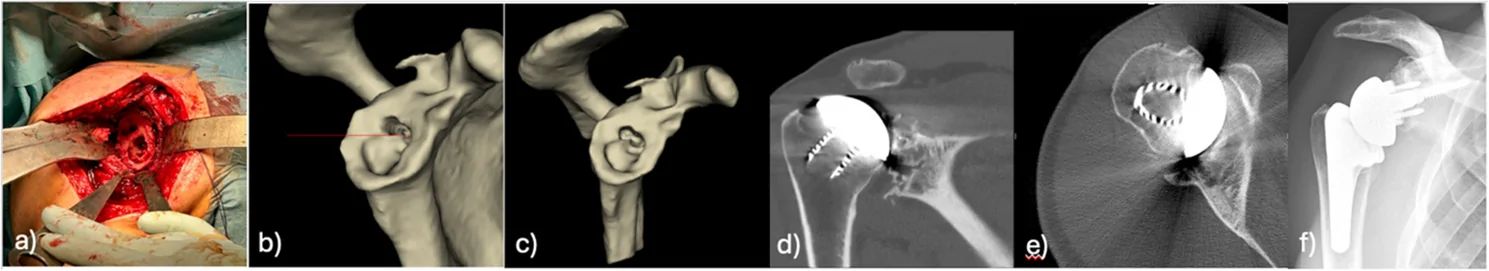
**a** intraoperative photograph of a contained glenoid defect (**b**) and (**c**) 3D reconstructed image from CT using Blueprint preoperative planning software (**d**) coronal 2D CT image (**e**) axial 2D CT image of the same contained defect (**f**) postoperative a.p.- X-ray of a reverse short-stem arthroplasty following a one-stage revision

**Fig. 2:**
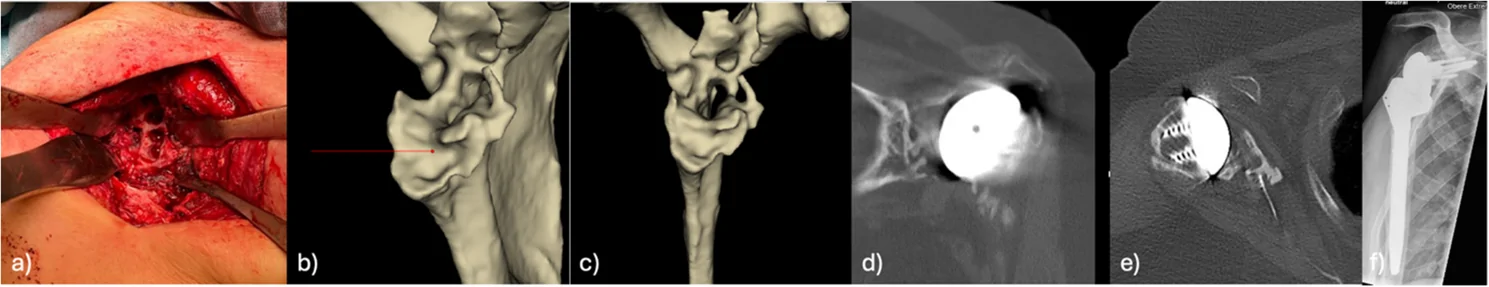
**a** intraoperative photograph of an uncontained glenoid defect (**b**) and (**c**) 3D reconstructed image from CT using Blueprint preoperative planning software (**d**) coronal 2D CT image (**e**) axial 2D CT image of the same uncontained defect (**f**) postoperative a.p.- X-ray of a reverse long-stem total shoulder arthroplasty following two-stage revision

Containment status was also assessed on preoperative imaging (3D-CT reconstructions and 2D-CT scans) by two independent fellowship-trained shoulder surgeons (Figs. 1 and 2).

### Data analyses

Descriptive statistics were used to summarize patient characteristics, including means and standard deviations for continuous variables and frequencies and percentages for categorical variables. Glenoid defect classifications according to Antuña and Williams-Iannotti were analyzed descriptively, reporting the frequency and relative proportion of each defect type. Inter-method agreement between 2D-CT, CT-derived 3D reconstructions, and intraoperative assessments of glenoid containment, as well as interobserver agreement, were evaluated using Cohen’s κ coefficient. Agreement was interpreted according to standard thresholds: κ > 0.80 indicated almost perfect agreement; 0.60–0.80, substantial; 0.40–0.60, moderate; 0.20–0.40, fair; and < 0.20, slight agreement. All analyses were performed using Stata/SE version 17.0 (StataCorp LLC, College Station, TX).

## Results

A total of 47 patients were included in this study. The mean age at the time of primary arthroplasty was 63 ± 9 years, and 25 patients (53%) were female (see detailed description in Table 1).

**Table 1:** Patient characteristics

Variable	
Female Sex, *N* (%)	25 (53)
Age (years), mean (SD)	62.7 (8.9)
Affected side, *N* (%)	
Right	29 (62)
Left	18 (38)
Surgery duration in minutes (primary), M (SD)	109.7 (25.9)
Surgery duration in minutes (revision), M (SD)	130.9 (44.8)
Primary Diagnosis, *N* (%)	
Rheumatoid Arthritis	1 (2)
Fracture sequelae	2 (4)
Primary Osteoarthritis	38 (81)
Primary Humeral Head Necrosis	1 (2)
Osteoarthritis due to Instability	5 (11)

The primary indication for arthroplasty was primary osteoarthritis (*n* = 38; 81%), followed by osteoarthritis due to instability (*n* = 5; 11%), and fracture sequelae (*n* = 2; 4%). The mean time from primary arthroplasty to revision was 7 ± 3 years. The most common indication for revision surgery was glenoid component loosening (*n* = 38; 79%), followed by prosthetic failure (*n* = 2; 4%), periprosthetic fracture (*n* = 1; 2%), and rotator cuff pathology (*n* = 1; 2%). Among the 47 revision procedures, 8 were performed as two-stage revisions.

### Glenoid defect classification

Glenoid bone loss in the 47 revision cases was classified using two established systems (Table 2).

**Table 2:** Frequency and percentage of glenoid bone loss categories by classification system (*N* = 47)

Category	Antuña *N* (%)	Iannotti *N* (%)
Not classifiable	3 (6.4)	0 (0)
Central moderate	1 (2.1)	-
Combined moderate	2 (4.3)	-
Combined severe	3 (6.4)	-
Mild central	13 (27.7)	-
Mild combined	2 (4.3)	-
Moderate central	10 (21.3)	-
Moderate combined	5 (10.6)	-
Severe central	2 (4.3)	-
Severe combined	6 (12.8)	-
S + R+V+		15 (31.9)
S + R+V-		2 (4.3)
S + R-V+		5 (10.6)
S-R + V+		6 (12.8)
S-R + V-		3 (6.4)
S-R-V+		7 (14.9)
S-R-V-		9 (19.2)

S^+^R^+^V^+^ → Superior pillar, retrocoracoid pillar, and vault are all intact; S^-^R^-^V^-^ ; All three structures are deficient; S^-^R^-^V^+^ ;Superior and retrocoracoid pillars are deficient, but the vault is preserved

According to Antuña et al., the most common patterns were mild central defects (27.7%) and moderate central defects (21.3%), followed by severe combined loss (12.8%). Three cases (6.4%) could not be classified using this specific system.

Based on the Iannotti vault-based classification, S⁺R⁺V⁺ was the most common configuration (31.9%), followed by S⁻R⁻V⁻ (19.2%) and S⁻R⁻V⁺ (14.9%).

### Containment assessment and agreement analysis

Based on intraoperative photographs or operative reports, 32 of 47 cases (68.1%) were classified as contained defects. Containment of glenoid defects was then assessed on preoperative 3D-CT and 2D-CT. Agreement between assessment modalities as well as interobserver reliability was evaluated using Cohen’s kappa coefficient. Agreement between 3D reconstructed images derived from CT and intraoperative findings was 80.9% (κ = 0.58; *p* < 0.001), indicating moderate agreement. On 3D-CT, 29 of 47 cases (61.7%) were classified as contained and 18 (38.3%) as uncontained. Agreement between 2D-CT and intraoperative assessment was 65.9% (κ = 0.37; *p* = 0.0015), representing fair agreement. On 2D-CT, 18 of 47 cases (38.3%) were considered contained and 29 (61.7%) uncontained (Table 3). Inter-rater agreement was strong for 2D (κ = 0.75) and near perfect for 3D (κ = 0.82), with both significantly exceeding chance agreement (*p* < 0.001).

**Table 3:** Containment classification and agreement analysis

Contrasts	Contained *N* (%)	Non- Contained *N* (%)	Total (*N*)	Agreement (%)	Kappa	Interpretation	*p*-value
3D-CT (Blueprint)	29 (61.7)	18 (38.3)	47	—	—	—	—
2D-CT	18 (38.3)	29 (61.7)	47	—	—	—	—
Intraoperative	32 (68.1)	15 (31.9)	47	—	—	—	—
3D-CT vs. Intraoperative	—	—	47	80.9%	0.58	Moderate agreement	< 0.001
2D-CT vs. Intraoperative	—	—	47	65.9%	0.37	Fair agreement	0.0015
2D-CT vs. 3D-CT	—	—	47	72.3%	0.48	Moderate agreement	0.0001
3D-CT: Interobserver reliability	—	—	47	91.5%	0.82	almost perfect agreement	< 0.001
2D CT: Interobserver reliability	—	—	47	87.2%	0.75	Substantial agreement	< 0.001

## Discussion

The primary aim of this study was to evaluate the accuracy of the Blueprint Revision 3D-CT planning software by comparing preoperative reconstructions with intraoperative findings. To our knowledge, this represents the first investigation to evaluate revision shoulder arthroplasty planning software in terms of its ability to correctly visualize containment of glenoid defects. Prior studies primarily focused on intraoperative classification systems, conventional radiographic or CT-based assessments, or reconstruction outcome series, rather than validating revision-specific 3D planning software against intraoperative containment [11, 13, 16–18].

In primary shoulder arthroplasty, 3D preoperative planning has demonstrated clear advantages over conventional 2D imaging, with multiple studies reporting improved characterization of glenoid wear, version, and inclination, ultimately informing implant selection and surgical outcomes [14, 15]. Although in our study agreement was moderate rather than substantial, 3D-CT consistently outperformed 2D imaging and may provide clinically useful additional information for preoperative planning.

Improved preoperative characterization of glenoid defect containment may have important implications for surgical planning in revision shoulder arthroplasty. More accurate identification of morphology of glenoid defects may support decisions regarding bone grafting strategies, implant selection, and overall reconstructive feasibility. In cases with advanced bone loss, accurate preoperative assessment may help determine whether definitive reconstruction is feasible in a single-stage procedure or whether staged management is more appropriate. Inaccurate assessment on conventional imaging may increase intraoperative uncertainty and necessitate unplanned changes in strategy.

For example in primary arthroplasties, 3D imaging reduces the risk of missed posterior wear and corrects under- or overestimation errors common with 2D CT, which can alter surgical planning [19]. Consistent with these findings, our results showed 2D-CT tended to underestimate containment, which may result in an overestimation of defect severity and consequently affect preoperative decision-making. In contrast, 3D reconstructed images derived from CT provided a more reliable depiction of glenoid morphology, supporting its utility as a clinically relevant tool for preoperative planning in revision shoulder arthroplasty. These advantages may translate into more precise surgical planning and may reduce the risk of overtreatment.

Metal artifacts and retained implants often obscure glenoid defects, degrading imaging quality and limiting accurate preoperative assessment. Our study primarily involved revisions of aTSA with all-polyethylene glenoid components, which generate substantially less metal artifact on preoperative CT imaging. Nevertheless, concordance between 2D CT assessment and intraoperative findings remained lower than that achieved with 3D reconstructed images derived from CT. This discrepancy may be even more pronounced in cases involving other implant types, such as metal-backed glenoid components, where metal artifact further degrades CT image quality and compromises accurate assessment of glenoid bone loss.

Collectively, our findings suggest that central defects were the predominant pattern of bone loss. For uncontained or atypical defects, unclassifiable cases demonstrate each classification system’s limitations. Since 3D visualization may improve complex defect morphology representation, more comprehensive and adaptable classification tools are needed. Specifically, existing classification systems cannot adequately represent the bone loss patterns associated with failed pegged glenoid components, which hinders surgical planning and reporting consistency.

Advanced imaging and 3D planning software are beneficial, but the technical workflow can be laborious for engineers. Manually identifying artifacts and foreign materials slice-by-slice in each CT scan prolongs the planning process and increases associated costs. In the future, automation, especially segmentation, can boost efficiency, lower costs, and increase accessibility.

Managing extended glenoid bone loss with alternative methods like patient-specific instrumentation (PSI) and customized implants is expensive, time-consuming, and depends on the preoperative CT scan. These methods can be limited by suboptimal imaging, processing, or scan-to-surgery time.

This study has several limitations. First, its retrospective design may have introduced selection and documentation bias, as only patients with complete imaging and surgical records were included. Second, intraoperative findings served as the reference standard; although they represent the most direct clinically available assessment of glenoid bone loss, they remain partly subjective and were based on retrospective review of operative reports and photographs. Third, postoperative clinical outcomes and comparisons with alternative planning strategies, such as patient-specific instrumentation or custom implants, were not evaluated, limiting conclusions regarding the clinical impact of improved imaging agreement. Fourth, the relatively small sample size and absence of an a priori sample size calculation limit the statistical power and generalizability of the findings. Finally, only a single planning platform was assessed, and CT segmentation was performed with support from engineers affiliated with the manufacturer, which may affect the external validity of the results. However, these individuals had no involvement in clinical assessments, statistical analysis, interpretation of the findings, or manuscript preparation. are needed to further validate the role of 3D planning in revision shoulder arthroplasty.

Future research should address these limitations through larger, prospective studies focusing on these comparisons and incorporate longitudinal clinical outcome data to substantiate the clinical significance of imaging-driven planning. Furthermore, prospective studies integrating intraoperative navigation or other quantitative registration methods may provide a more objective reference standard for validating advanced imaging technologies.

Enhancing preoperative glenoid bone stock evaluation reduces intraoperative complications, optimizes implant selection, and may reduce operative time and risks. Future initiatives must prioritize the automation of segmentation workflows, the refinement or expansion of classification frameworks, and the assessment of the clinical impact of 3D preoperative planning. Improvements are crucial for enhancing the reliability, efficiency, and standardization of surgical planning in the evolving field of shoulder arthroplasty.

## Conclusion

The clinical relevance of this study lies in demonstrating that 3D-CT planning software provides a preoperative assessment of glenoid defect containment that demonstrated a higher agreement with intraoperative findings compared to conventional 2D-CT. The software facilitates comprehensive preoperative classification of glenoid bone loss. 3D planning tools offer good assistance in complex revision shoulder arthroplasty by minimizing intraoperative uncertainty and aiding in implant selection. These results support the potential value of 3D virtual planning software in revision shoulder arthroplasty; however, broader clinical application may be constrained by factors such as the manual processing of CT scans, susceptibility to imaging quality, and associated costs.

## Data Availability

The datasets generated and analyzed during the current study are available from the corresponding author on reasonable request.

## Abbreviations

2D: Two–dimensional
2D: CT–Two–dimensional computed tomography
3D: Three–dimensional
3D: CT–Three–dimensional computed tomography
aTSA: Anatomic total shoulder arthroplasty
CT: Computed tomography
IRB: Institutional Review Board
KEK: ZH–Cantonal Ethics Committee Zurich
MI: Michigan
N: Number
SD: Standard deviation
SE: Standard error
PSI: Patient–specific instrumentation
TX: Texas
USA: United States of America
κ(kappa): Cohen’s kappa coefficient
